# Quantitative MRI for analysis of peritumoral edema in malignant gliomas

**DOI:** 10.1371/journal.pone.0177135

**Published:** 2017-05-23

**Authors:** Ida Blystad, J. B. Marcel Warntjes, Örjan Smedby, Peter Lundberg, Elna-Marie Larsson, Anders Tisell

**Affiliations:** 1 Department of Radiology and Department of Medical and Health Sciences, Linköping University, Linköping, Sweden; 2 Centre for Medical Image Science and Visualization (CMIV), Linköping University, Linköping, Sweden; 3 Division of Cardiovascular Medicine, Department of Medical and Health Sciences, Linköping University, Linköping, Sweden; 4 School of Technology and Health, KTH Royal Institute of Technology, Stockholm, Sweden; 5 Department of Radiation Physics and Department of Medical and Health Sciences, Linköping University, Linköping, Sweden; 6 Department of Surgical Sciences, Radiology, Uppsala University, Uppsala, Sweden; University of Chicago, UNITED STATES

## Abstract

**Background and purpose:**

Damage to the blood-brain barrier with subsequent contrast enhancement is a hallmark of glioblastoma. Non-enhancing tumor invasion into the peritumoral edema is, however, not usually visible on conventional magnetic resonance imaging. New quantitative techniques using relaxometry offer additional information about tissue properties. The aim of this study was to evaluate longitudinal relaxation R_1_, transverse relaxation R_2_, and proton density in the peritumoral edema in a group of patients with malignant glioma before surgery to assess whether relaxometry can detect changes not visible on conventional images.

**Methods:**

In a prospective study, 24 patients with suspected malignant glioma were examined before surgery. A standard MRI protocol was used with the addition of a quantitative MR method (MAGIC), which measured R_1_, R_2_, and proton density. The diagnosis of malignant glioma was confirmed after biopsy/surgery. In 19 patients synthetic MR images were then created from the MAGIC scan, and ROIs were placed in the peritumoral edema to obtain the quantitative values. Dynamic susceptibility contrast perfusion was used to obtain cerebral blood volume (rCBV) data of the peritumoral edema. Voxel-based statistical analysis was performed using a mixed linear model.

**Results:**

R_1_, R_2_, and rCBV decrease with increasing distance from the contrast-enhancing part of the tumor. There is a significant increase in R_1_ gradient after contrast agent injection (P < .0001). There is a heterogeneous pattern of relaxation values in the peritumoral edema adjacent to the contrast-enhancing part of the tumor.

**Conclusion:**

Quantitative analysis with relaxometry of peritumoral edema in malignant gliomas detects tissue changes not visualized on conventional MR images. The finding of decreasing R_1_ and R_2_ means shorter relaxation times closer to the tumor, which could reflect tumor invasion into the peritumoral edema. However, these findings need to be validated in the future.

## Introduction

High-grade malignant gliomas are primary brain tumors with an incidence of ≈ 5/100, 000. The prognosis is poor with a median survival of approximately 12–15 months [1]. Magnetic Resonance Imaging (MRI) is extensively used in the diagnostic work-up as well as to monitor treatment. At diagnosis, these tumors show heterogeneous contrast enhancement, often with a necrotic center, and peritumoral edema. Conventional MR imaging relies mainly on visual assessment and the neuroradiologists’ ability to recognize patterns. Tumors show contrast enhancement on T1-weighted images (T1W) after gadolinium based contrast agent injection, and the peritumoral edema has a high signal on T2-weighted/fluid attenuation inversion recovery images (T2W/FLAIR). However, it is well known that high-grade malignant gliomas extend beyond the contrast-enhancing border. Diffuse non-enhancing tumor infiltrates into the peritumoral edema [2], and the non-enhancing portions of the tumor have to be considered when assessing patients during follow-up [3]. These diffuse tumor-associated tissue changes are difficult to detect visually on conventional images, and hence the commonly used physiological MR sequences diffusion- and perfusion-weighted imaging (DWI and PWI) may add quantitative information about tumor quality and extension [4]. Diffuse, non-enhancing tumor infiltration into the peritumoral edema can be assessed by a gradient in apparent diffusion coefficient (ADC) values due to the higher cell density [5]and increased cerebral blood volume (CBV) that reflect the tumor-induced neoangiogenesis [6].

High-grade malignant gliomas are aggressive tumors, and they receive aggressive treatment with surgery, chemotherapy, and radiation. This treatment can cause secondary reactions in the non-tumorous brain tissue, resulting in edema and contrast enhancement, i.e. a pattern similar to tumor growth [7,8], which complicates the assessment of follow-up examinations. In these cases with treatment-related changes, such as pseudoprogression and radiation necrosis, conventional MR imaging does not suffice, and quantitative methods, such as DWI, PWI, and MR spectroscopy can add information that can aid in the differentiation between treatment-related changes and tumor recurrence [9–13]. Studies of the peritumoral region with multiparametric pattern analysis to detect the infiltrating parts of the tumor to predict possible locations of tumor recurrence have demonstrated heterogeneity of the peritumoral region [14]. Computational analysis of MR perfusion has provided similar results [15]. However, these methods require advanced mathematical modeling and are not easily implemented in the clinical workflow. So, despite the current arsenal of quantitative/semi-quantitative techniques, follow-up of malignant gliomas remains a challenge because results of the existing methods can be difficult to interpret or to implement in the clinical setting. Hence, there is an interest and a need for development of clinically applicable and robust quantification methods when evaluating patients with brain tumors, in the diagnostic work-up as well as for treatment evaluation and follow-up.

Synthetic MRI [16] is a quantitative MR- sequence (qMRI), acquired in approximately 6 minutes to obtain proton density (PD), longitudinal relaxation rate (R_1_) and transverse relaxation rate (R_2_), with correction for B_1_- inhomogeneity. From this sequence, synthetic images with a free range of weightings can be produced. The quantitative information of the sequence enables relaxometry measurements of the tissue, which could be a useful addition to conventional imaging in brain tumor investigations.

The purpose of this study was to analyze the relaxation properties of the peritumoral edema in patients with malignant gliomas before surgery, using SyMRI to assess whether relaxometry can detect changes not visible on conventional images of the peritumoral edema.

## Materials and methods

### Subjects

Twenty-four patients with typical radiological findings suggestive of a high-grade malignant glioma were prospectively included in the study from 2013 to 2016 and examined with MRI before surgery. The diagnosis was confirmed by histopathological analysis after surgery. For patient demographics, see Table 1. Three patients with a histopathological diagnosis of lymphoma, abscess, and primitive neuroectodermal tumor (PNET) were excluded. Two patients were not analyzed due to difficulties in delineating the contrast- enhancing part of the tumor from the peritumoral edema.

**Table 1:** Patient demographics.

Patient	Sex	Age	Diagnosis
**1**	M	63	Anaplastic oligodendroglioma III
**2**	M	71	Glioblastoma
**3**	F	58	Glioblastoma
**4**	M	63	Glioblastoma
**5**	F	57	Glioblastoma
**6**	M	65	Glioblastoma
**7**	F	61	Glioblastoma
**8**	F	65	Anaplastic oligodendroglioma III
**9**	M	69	Glioblastoma
**10**	M	34	Anaplastic oligodendroglioma III
**11**	M	50	Anaplastic oligodendroglioma III
**12**	M	79	Glioblastoma
**13**	M	68	Glioblastoma
**14**	M	71	Glioblastoma
**15**	M	65	Gliosarcoma
**16**	M	46	Glioblastoma
**17**	M	72	Glioblastoma
**18**	M	76	Glioblastoma
**19**	F	45	Glioblastoma

The local institutional review board approved the study, and informed written consent was obtained from all patients. Ethical approval was obtained from the Regional Ethical Board Linköping, Sweden, decision number 2011/406-31.

### MR acquisition

Images were acquired on a three-tesla (3T) MR scanner (750, GE Medical Systems, Milwaukee, Wisconsin) using a 32-channel phased array head coil according to our clinical protocol for brain tumor investigation, with the addition of the quantitative MR sequence SyMRI MAGIC. The clinical protocol for brain tumor investigation consists of axial T2WI-FLAIR, T1WI, T2WI, dynamic susceptibility contrast (DSC) perfusion, T1WI-GD, 3D-FSPGR (fast spoiled gradient echo) Gd, and DWI. The sequence parameters for the conventional images used in the study analysis were as follows:

**DSC Perfusion gradient-echo EPI (echo planar imaging)**; axial, field of view (FOV) 220 × 165, 1 680 slices, voxel size 1.7 × 1.7 × 5 mm (gap 1 mm), TE (echo time) = 29 ms, TR (repetition time) = 1 340 ms, flip angle 60. The perfusion had a 6 s delay before injection with a standard contrast dose of 10 ml gadopentetate dimeglumine (Gd-DTPA, Omniscan, GE Healthcare) 0.5 mmol/ml with an injection rate of 4 ml/s followed by a 15 ml saline flush at an injection rate of 4 ml/s. The total amount of contrast agent was 0.2 ml/kg, with the remaining dose injected after the perfusion series.

**T1W spin echo before and after (T1WGd) contrast agent injection**: axial, FOV 220 × 165, 24 slices, voxel size 0.43 × 0.43 × 5 mm (gap 1 mm), TE = 17.7 ms, TR = 2 524 ms, TI (inversion time) = 798 ms.

**T2W spin echo PROPELLER**: axial, FOV 220 × 220, 24 slices, voxel size 0.43 × 0.43 × 5 mm (gap 1 mm), TE = 95–97 ms, TR = 3 000 ms.

The quantitative sequence [16], is a multi-slice, multi-echo and multi-saturation delay qMRI technique for simultaneous measurement of R_1_, R_2_, and PD, with the following parameters in this study.

**qMRI MAGIC**; axial, FOV 220 × 180, 24 slices, voxel size 0.43 × 0.43 × 5 mm (gap 1 mm). In total 8 images per slice were measured with TE = 22 ms or 95 ms, TR = 4 000 ms, TI = 170, 670, 1840 or 3840 ms. The scan time was 5:55 minutes, and the qMRI-series was obtained before and after contrast agent injection.

### Post-processing and ROI placement

#### qMRI post-processing

**Fig 1:**
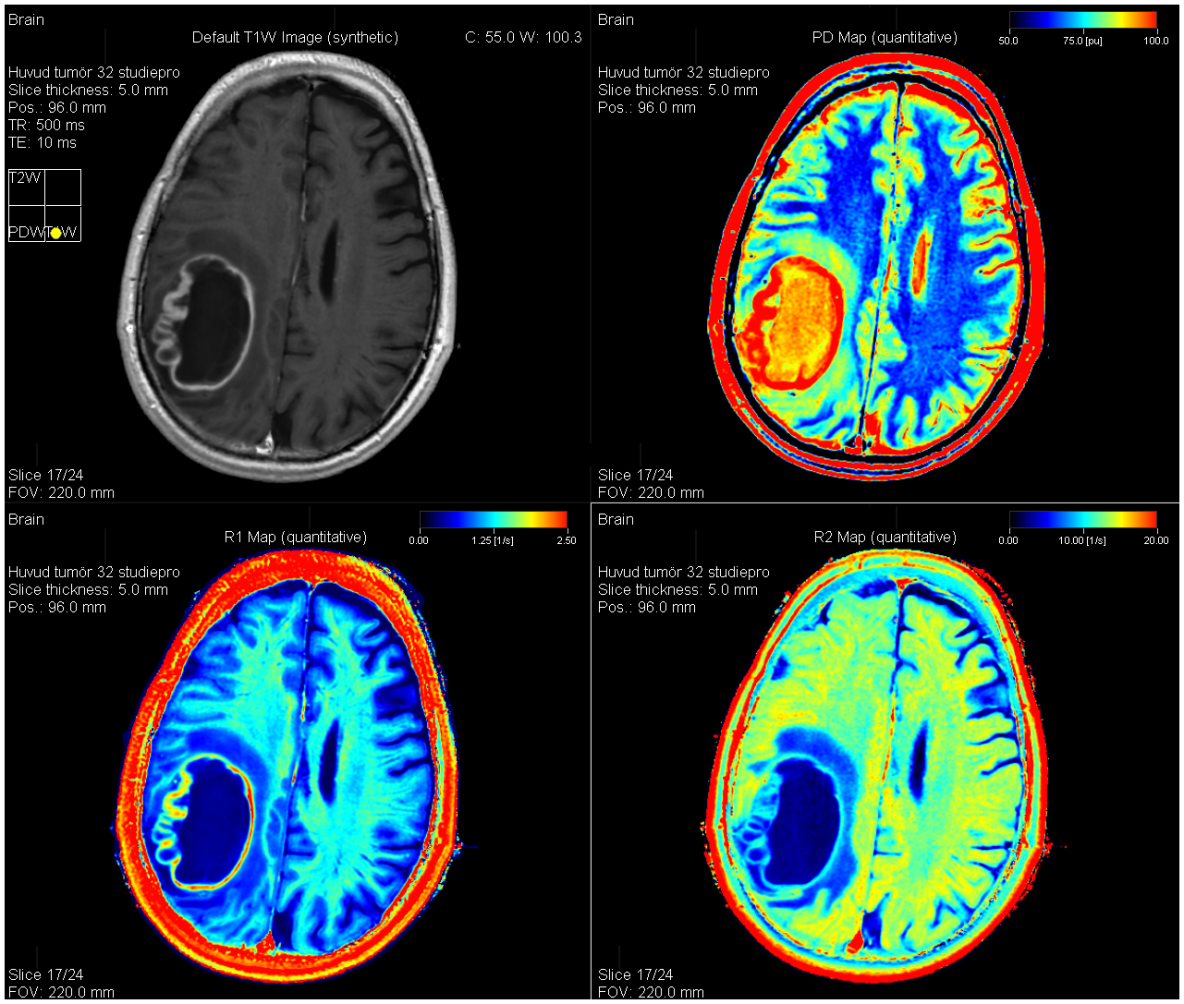
Synthetic images created from the quantitative scan. An example of synthetic T1GdWI (top left), proton density map (top right), R_1_ map (bottom left), and R_2_ map (bottom right) in a 68-year-old male with a glioblastoma.

The qMRI sequence yields quantitative maps of R_1_, R_2_, and PD, which are used for measurements and to create synthetic images matching the conventional images (Fig 1).

The post-processing time of the raw image dataset was approximately 1 minute on an ordinary PC using SyMRI 8 software (SyntheticMR AB, Linköping, Sweden) to create the synthetic images. Relaxation time values (T-values) were obtained from R_1_ and R_2_ by calculating T = 1/R.

#### Perfusion post-processing

The perfusion data were transferred to a workstation and analyzed with the software NordicICE (version 2.3.12, NordicNeuroLab AS, Bergen, Norway). After motion correction, the realigned perfusion data were used to create whole-brain CBV maps with auto- detected noise threshold and leakage correction. CBV values were normalized against the contralateral hemisphere to obtain relative CBV (rCBV).

#### ROI analysis

Conventional T2W, T1W, and T1W-Gd images and corresponding synthetic images were transferred to the software MevisLab version 2.7 (MeVis Medical Solutions AG, Bremen, Germany).

**Fig 2:**
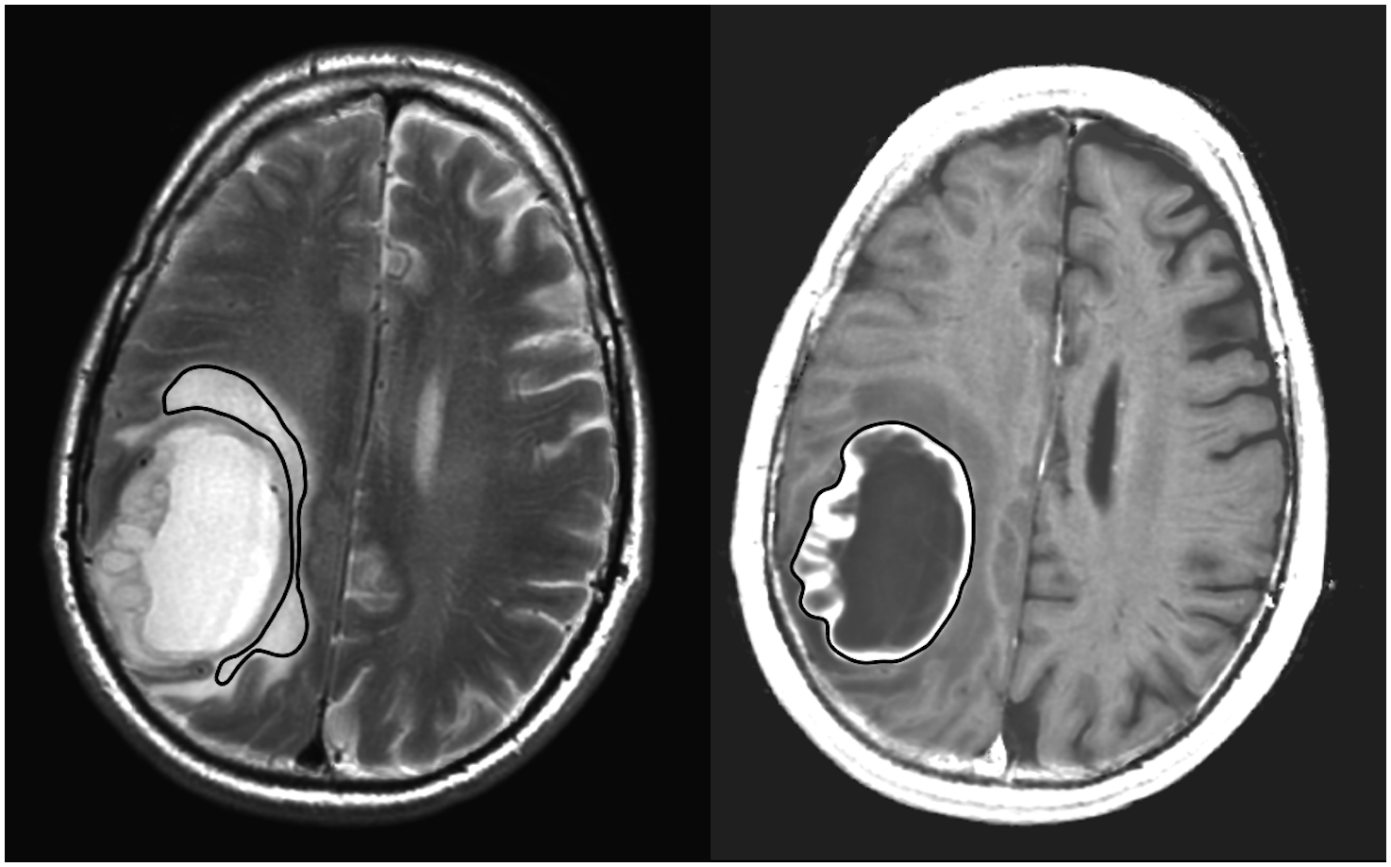
ROIs delineating the peritumoral edema and the contrast-enhancing part of the tumor. A ROI was placed in the peritumoral edema of the synthetic T2WI. The edema ROI was then applied in the qMRI volumes to obtain the quantitative values of R_1_, R_2_, and proton density. The contrast-enhancing part of the tumor was manually delineated in the synthetic T1GdWI and subtracted to avoid overlap with the edema ROI.

A neuroradiologist drew regions of interest (ROIs) for the analysis. The contrast-enhancing border of the tumor was delineated manually (tumor-ROI) in the synthetic T1W image (Fig 2), and a free-hand ROI (edema-ROI) was drawn in the peritumoral edema outside the contrast-enhancing part of the tumor in the synthetic T2W image (Fig 2). ROIs were also placed in synthetic T2W images in the normal appearing white matter (NAWM) adjacent to the tumor edema (NAWM-near-Tumor-ROI), and in the corresponding lobe in the contralateral hemisphere (NAWM-contra-ROI).

ROIs delineated in synthetic T2W image volume were directly applied to all images, and to qMRI-maps that were calculated from the same qMRI volume.

However, to apply the tumor-ROI delineated in the qMRI volume post-gadolinium in the qMRI volume pre-gadolinium, a coordinate transformation matrix, M_GD2pre_, was calculated by image registration of the synthetic T2W-Gd volume to the synthetic T2W volume. The image registration was performed using MevisLab MERIT module, with registration method set to ‘‘3D rigid” and similarity measurement to ‘‘SSD”. To avoid overlap the tumor-ROI was subtracted from the edema-ROI. The inverse transformation matrix M_GD2pre_^-1^ was used to transform the edema-ROI, NAWM-near-Tumor-ROI and NAWM-contra-ROI to the qMRI-Gd volume.

The edema-ROI, NAWM-near-Tumor-ROI, and NAWM-contra-ROI were also transformed to the CBV map of the perfusion analysis to obtain the rCBV values of the peritumoral edema. This transformation matrix was also calculated using image registration. The synthetic T2W volume was registered to the baseline EPI of the perfusion series using Mevis Lab MERIT module, with the registration method set to 3D rigid transformation and rescaling in each direction and using the normalized cross-correlation (NCC) for similarity measurement.

### Statistics

First, the mean values for R_1_, R_2_, PD and rCBV for each individual ROI were calculated. These values were used to obtain the group mean and standard deviation of each ROI type (edema-ROI, NAWM-near-Tumor-ROI, and NAWM-contra-ROI).

In the edema-ROI, the relationship between the quantitative values and the distance of each individual voxel to the contrast-enhancing part of the tumor was investigated using mixed linear models. The voxel values of the R_1_, R_2_, PD, and rCBV values were used as dependent variables, the distance to the tumor-ROI was treated as fixed effect, and subject was treated as a random effect.

The difference in slope for R_1_ post-Gd compared to the slope in R_1_ pre-Gd was analyzed using Student’s *t*- test.

All statistical calculations were performed in JMP 8.0 (SAS Inc, Cary, North Carolina).

## Results

**Fig 3:**
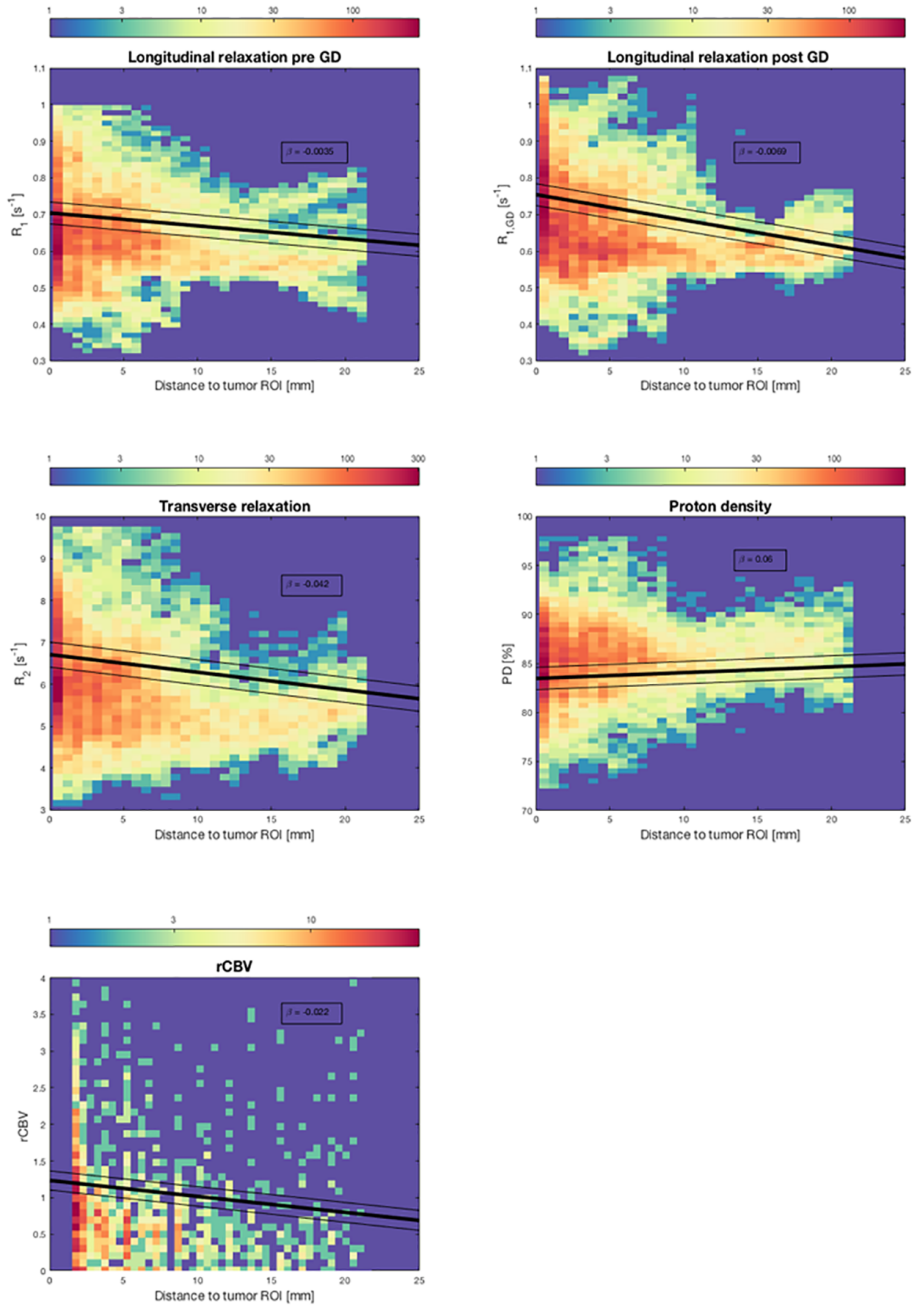
Color histograms of R_1_, R_2_, proton density and rCBV. Color histograms of R_1_, R_2_, proton density, and rCBV in the peritumoral edema in relation to distance from the contrast-enhancing part of the tumors of all patients. The thick black lines represent the regression line given by the mixed linear models, with the slope presented as beta value in the figure. The thin black lines represent the confidence intervals. For R_1_, R_2_, and rCBV, there is a decrease in values with increasing distance from the contrast-enhancing part of the tumor. Proton density has a slight decrease with increasing distance. There is a significant increase in the gradient of the relaxation values after contrast agent injection (*p* < 0.001). The pattern of relaxation values is more heterogeneous closer to the contrast-enhancing part of the tumor.

Measurements of relaxation values in the peritumoral edema revealed a decrease in R_1_ and R_2_ with increasing distance to the contrast-enhancing border of the tumor, with a significant (*P*<0.0001) gradient from the contrast-enhancing border to the periphery of the edema (Fig 3), with the slope presented as beta-value in the figure. There was a slight increase in PD with increasing distance from the contrast-enhancing part of the tumor. After gadolinium-based contrast agent injection, there was a significant increase in gradient in R_1_ (*P* < .0001) (Fig 3).

As seen in Fig 3, there is a heterogeneous pattern of relaxation values within the first 10 mm of the peritumoral edema, possibly reflecting non-enhancing infiltrating tumor as well as peritumoral edema.

rCBV values were higher closer to the tumor, with a gradient from the contrast-enhancing border of the tumor to the periphery of the peritumoral edema (Fig 3).

**Fig 4:**
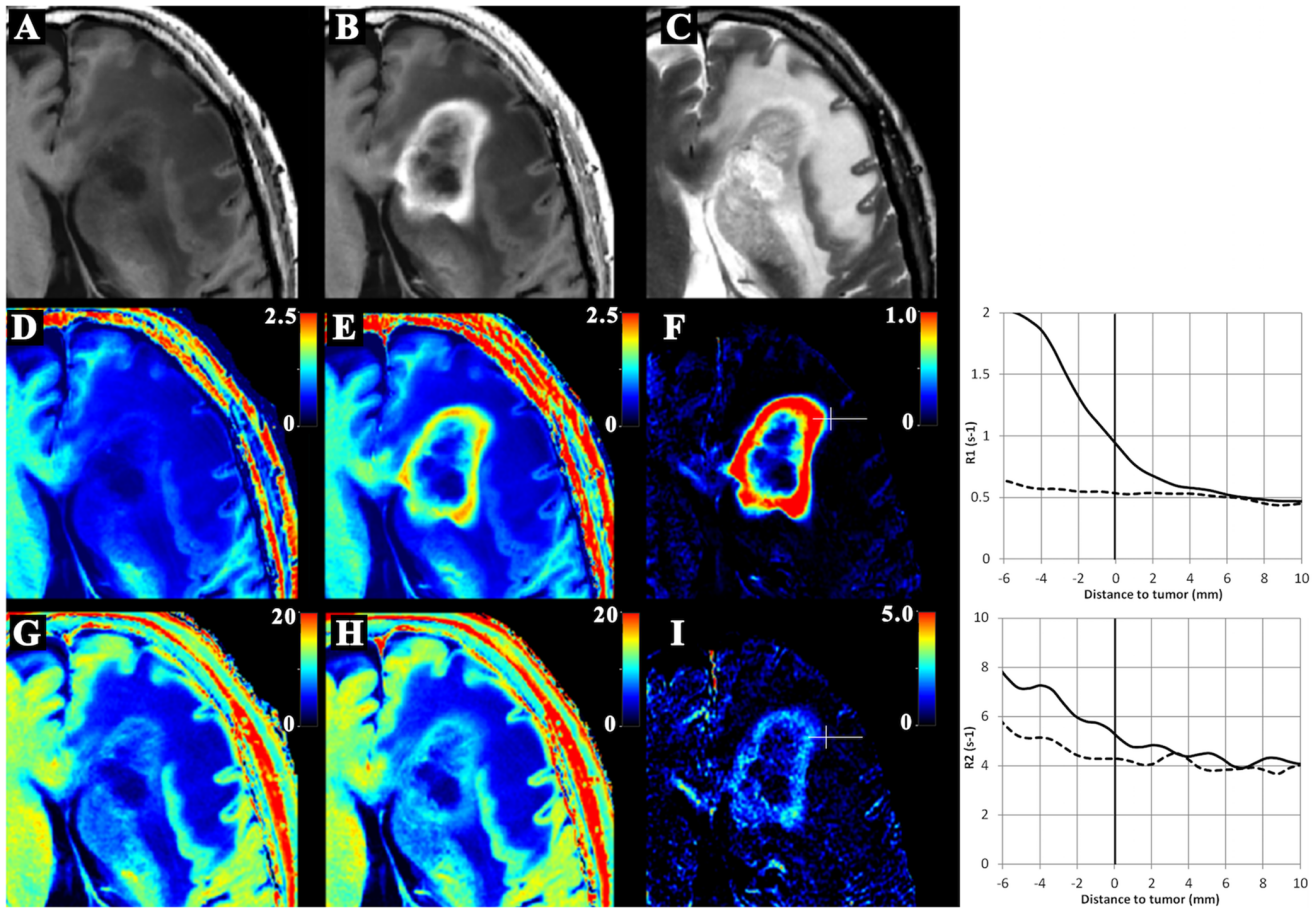
An example of synthetic MRI in one of the patients. Fig 4 is an example of the synthetic and quantitative images of a 76-year-old man with glioblastoma. The top row shows the synthetic images: (A) native T1WI, (B) post-GD T1WI, (C) T2WI post-GD. The center row shows the R1 maps: (D) native R1 map, (E) post-GD R1 map, (F) difference-map of post-GD R1 minus native R1. The bottom row shows the R2 maps: (G) native R2 map, (H) post-GD R2 map, (I) difference-map of post-GD R2 minus native R2. In (F) and (I), a white line is indicated, along which native (dotted line) and post-GD (solid line) data are plotted regarding R1 (top) and R2 (bottom) as a function of the distance to tumor, as shown in the diagrams. The zero distance point is indicated by the small perpendicular line in (F) and (I).

Fig 4 is an example of synthetic MR images, quantitative maps and graphs of R_1_ and R_2_ in one of the patients, a 76-year-old man with a glioblastoma. The R_1_ graph shows an increase of R_1_ after gadolinium based contrast agent injection, with a ‘‘tail” extending out into the peritumoral edema. The R_2_ graph also depicts a gradient extending into the peritumoral edema.

Table 2 shows the relaxation times, PD, and rCBV of the different tissues measured (edema, NAWM-near-tumor, and NAWM-in-contralateral-hemisphere). Table 3 depicts the slopes with the intercept for the qMRI values and rCBV in relation to distance to the contrast-enhancing part of the tumor.

**Table 2:** qMRI values of ROIs: Group mean and SD (N = 19).

Tissue		R1(s^-1^)	R2(s^-1^)	PD(%)	rCBV
		Mean ± SD	Mean ± SD	Mean ± SD	Mean ± SD
**Edema-ROI**	Pre-Gd	0.69 ± 0.13	6.5 ± 1.3	84 ± 5	
	Post-Gd	0.72 ± 0.14	6.8 ± 1.4	82 ± 6	1.1 ± 0.6
**NAWM-near- tumor-ROI**	Pre-Gd	1.32 ± 0.11	12.8 ± 0.9	66 ± 3	
	Post-Gd	1.33 ± 0.11	12.9 ± 0.9	66 ± 3	1.2 ± 1.2
**NAWM-contra-ROI**	Pre-Gd	1.30 ± 0.10	12.5 ± 1.0	66 ± 3	
	Post-Gd	1.31 ± 0.11	12.5 ± 1.1	65 ± 3	-

**Table 3:** Mixed linear model with qMRI values as dependent variable and distance to tumor as fixed effect.

	Intercept	Beta	t-value	R^2
**R_1_**	0.70	-0.0035***	-36	0.65
**R_1_GD**	0.75	-0.0069***	-53	0.53
				
**R_2_**	6.71	-0.042***	-41	0.69
**R_2_GD**	6.94	-0.033***	-28	0.63
				
**PD**	83.46	0.06***	14	0.50
**PDGD**	81.39	0.13***	25	0.48
				
**rCBV**	1.24	-0.02***	-4	0.2

Table 3 shows the intercept and the slope (beta) of the gradients and the significance levels.

*** = p < .001

## Discussion

In this study, we found a gradient of relaxation values in the peritumoral edema of malignant gliomas, with shorter relaxation times and a greater heterogeneity in the edema closest to the contrast-enhancing part of the tumor and an increase in the R1 gradient in the edema after gadolinium based contrast agent injection (Fig 3). These changes could indicate diffuse, non-enhancing tumor infiltration, not visible on conventional MR images.

The diffuse, non-enhancing tumor infiltration is difficult for the radiologist to detect but clinically important for the neurosurgeon and radiotherapy oncologist. During surgery, the aim is radical resection of the tumor [17]. In high-grade gliomas, radical resection is assessed based on the absence of residual contrast enhancement on the immediate post-surgical MRI follow-up performed within 48 hours of surgery. If radical resection is impossible due to tumor location near eloquent regions of the brain, the aim is maximal tumor resection with preservation of brain function. The extent of resection influences the prognosis, and it is known that malignant gliomas extend beyond their contrast-enhancing border with a diffuse, non-enhancing infiltration into the peritumoral edema, which complicates the assessment of the remaining tumor burden [18,19]. These diffuse, non-enhancing tissue changes are not easily detected visually on conventional MR images, but can to some extent be identified with diffusion and perfusion imaging, which correlates with histopathological features of the tumors [20]. While the diffusion is dependent on the Brownian motion of water and related to e.g. cell density in tumors, relaxation also depends on chemical interaction of the tissues on a microscopic level due to presence of lipids, proteins, macromolecules, and paramagnetic substances [21]. Relaxometry thus adds another type of quantitative information about the tissue.

Non-enhancing tissue changes may affect the outcome in patients with high-grade gliomas [17,22,23], with resection of non-enhancing tumor correlating with longer progression-free survival and overall survival [24].

Previous studies using relaxometry have shown that quantitative T2’ is associated with glioma grade, high grade gliomas (WHO III-IV) having a lower T2’ value than low-grade gliomas, possibly due to hyper-metabolism [25]. Other studies show that quantitative T1 and T2 mapping during follow-up of treated glioblastomas can provide earlier detection of tumor progression than conventional MR imaging, with a prolongation of T1 and T2 relaxation before the appearance of visible changes in conventional MR-images. The prolongation of T1 possibly results from subtle blood-brain-barrier damage not yet visible on the structural images [26,27]. Ellingson et al [28] used a T2 mapping technique to quantify edema reduction in recurrent glioblastoma treated with bevacizumab, and their result suggested a correlation between post-treatment T2 values and progression free survival.

Thus several studies indicate that measurement of T1 and T2 provides quantitative information about the non-visible tissue changes associated with gliomas, which could help elucidate the nature of these tumors, aid in their diagnostics, and assist treatment planning.

In the present study, we analyzed preoperative MRIs in a cohort of patients with malignant gliomas, currently being monitored during treatment, and future studies will analyze the follow-up examinations to assess treatment-related changes and tumor recurrence using synthetic MRI.

A limitation to this study is the lack of histopathological correlation of the relaxometry measurements of the infiltrated peritumoral edema. However, the findings are in line with other studies showing tumor extension beyond radiological borders in gliomas [29]. Even though the tumor was manually segmented from the peritumoral edema to avoid overlap, the accuracy of the ROI measurements might have been improved by a quantitative sequence with isotropic voxels and thinner slices, a technique that is currently under development.

The R^2^ values of the regression lines are relatively low, which indicates that the edema is complex, with several components. This is more evident closer to the contrast-enhancing border of the tumor, where the relaxation values have a heterogeneous pattern. The heterogeneous pattern could be related to the infiltration of tumor cells into the peritumoral edema.

## Conclusion

Quantitative T1 and T2 mapping can detect tissue changes in the peritumoral region that are not visible on conventional MR images. Relaxation values in the peritumoral edema have a heterogeneous pattern within the first 10 mm from the contrast-enhancing portion of the tumor with a gradient in relaxation values from the contrast-enhancing part of the tumor into the peritumoral edema. This may reflect non-visible tumor infiltration into the surrounding tissue, and this information could be useful for the planning of surgery and radiation therapy. The findings, however, need to be further validated.

## Supporting information

S1 Dataset Supporting information file with the quantitative values from the measurements. (ZIP)
